# The Dynamic Relationship between Innate Immune Biomarkers and Interferon-Based Treatment Effects and Outcome in Hepatitis C Virus Infection Is Altered by Telaprevir

**DOI:** 10.1371/journal.pone.0105665

**Published:** 2014-08-28

**Authors:** David F. G. Malone, Karolin Falconer, Ola Weiland, Johan K. Sandberg

**Affiliations:** 1 Center for Infectious Medicine, Department of Medicine Huddinge, Karolinska Institutet, Stockholm, Sweden; 2 Unit of Infectious Diseases, Karolinska University Hospital Huddinge, Stockholm, Sweden; University of Hawaii Manoa, United States of America

## Abstract

Soluble CD14 (sCD14) and IL-18 are markers and mediators of the innate immune response, and their plasma levels candidate biomarkers of HCV treatment effects and outcome. Here, we retrospectively studied sCD14 and IL-18 over the course of interferon-based treatment of HCV genotype 1 infection, with the aim to investigate the impact of direct-acting antivirals (DAAs) on the dynamics and relationships between these biomarkers and treatment effects and outcome. Two cohorts were followed longitudinally; one treated with standard dual therapy of pegylated IFNα and ribavirin, and one cohort receiving triple therapy including Telaprevir. sCD14 and IL-18 were measured before and during treatment and analyzed in relation to treatment effects. The initial analysis confirmed two patterns previously observed in patients with HCV/HIV-1 co-infection: Baseline levels of sCD14 were significantly lower in patients that went on to clear HCV infection in response to IFNα and ribavirin, and sCD14 levels were strongly induced during the course of this treatment. Interestingly, baseline levels of sCD14 and IL-18 in combination predicted treatment outcome in dual therapy better than either marker alone. Notably, these associations were weaker with the addition of Telaprevir to the treatment regimen, suggesting that the relationships between innate immune activation and outcome were altered and diminished by inclusion of a DAA in the treatment. In triple therapy, the dynamic increase of sCD14 in response to treatment was higher in patients clearing the virus, suggesting that the innate response to interferon is still significantly associated with outcome in patients treated with DAA-containing regimens. These results support the notion that levels of innate immune activation before and during treatment are associated with interferon-based treatment outcome. Furthermore, the addition of Telaprevir significantly alters the dynamics and relationships between innate immune biomarkers and treatment effects and outcome.

## Introduction

Chronic Hepatitis C virus (HCV) infection is treated to prevent progression to cirrhosis and development of hepatocellular cancer. Until recently the standard of care for treatment of HCV has consisted of a combination of pegylated interferon α (peg-IFNα) and ribavirin, which clears the virus in a significant fraction of patients, but at a high cost in terms of side effects. IFNα mobilizes a number of antiviral mechanisms, such as enhancement of MHC class I-mediated antigen presentation, activation of innate cellular immunity and increased transcription of IFN-stimulated genes [1]–[5]. The addition of first generation HCV protease inhibitors to peg-IFNα and ribavirin enhances response rates, leading the way towards substantial improvement in treatment of HCV disease [6]–[8]. New direct acting antivirals (DAAs) are revolutionizing the treatment of HCV infection. However, the use of peg-IFNα continues until the efficacy, safety, and cost effectiveness of IFN-free treatments have been fully clarified [1], [9]–[11]. How the addition of DAAs to IFNα-based therapy may alter relationships between innate immune responses and biomarkers on one hand, and treatment effects and outcome on the other, is largely unknown.

Soluble CD14 (sCD14) and IL-18 are two biomarkers of innate immune activation associated with viral disease. sCD14 is often considered as a marker of monocyte activation in response to lipopolysaccharide [12], and as a marker of microbial translocation [13], [14]. However, elevated levels of sCD14 have also been observed in patients with non-alcoholic steatohepatitis [15], and common variable immunodeficiency [16], [17], suggesting other possible origins for sCD14 in plasma, one of which may be the liver [18]–[20]. Interestingly, a high pre-treatment level of sCD14 is a negative predictor of virological response to peg-IFNα and ribavirin therapy in HCV/HIV-1 co-infected patients [21], [22].

IL-18 can be produced by macrophages and Kupffer cells [23], and plays a role in activation of NK cells and of T cell helper type I responses, both of which are integral components of the antiviral immune response. Known as a mediator and marker of inflammation, increased levels of IL-18 have been associated with chronic viral infections such as Epstein-Barr virus [24], and implicated in apoptosis of hepatocytes [25]. Levels of IL-18 are elevated in chronic HCV infection and associated with disease severity [26]–[28]. High IL-18 has also been observed in non-alcoholic fatty liver disease indicating a role and importance as biomarker of liver disease beyond HCV [29].

Given the role of sCD14 and IL-18 as markers and mediators of the innate immune response, their plasma levels and dynamics are candidate biomarkers of HCV treatment effects and outcome. In the present study, we evaluated the plasma levels of sCD14 and IL-18 over the course of treatment of HCV genotype 1 infection. Two groups of patients were retrospectively followed longitudinally, one group treated with standard dual therapy with IFNα and ribavirin alone, and one group receiving triple therapy adding Telaprevir to the standard treatment, with the goal to determine the dynamics of innate immune activation in relation to outcome during these treatments. The results indicate that baseline levels, as well as the on-treatment dynamics, of innate immune activation as measured by plasma sCD14 and IL-18 are associated with treatment outcome. Furthermore, the results suggest that the relationships between innate immune activation and treatment outcome are altered by introduction of a DAA in the treatment regimen.

## Patients and Methods

### Study cohort and samples

A retrospective study of 161 individuals infected with HCV genotype 1 was performed. Plasma levels of IL-18 and sCD14 were measured before and during treatment (Table 1). 72 patients were treated with standard peg-IFNα and ribavirin dual therapy, and out of these 71% were men, 79.2% were infected with genotype 1A, and 19.4% with genotype 1B. The median viral load was 1,900,000 IU/ml, and the sustained virological response (SVR) rate was 62.5%. Biopsy fibrosis scores were available for 68% of the patients in this group, and the majority of those exhibited fibrosis stage 2–3. A second group of 89 patients received triple therapy comprising peg-IFNα, ribavirin, and Telaprevir. In this group, 66% were men, 78.7% were infected with genotype 1A virus, 19.1% with genotype 1B, with a median viral load of 24,562 IU/ml, and the SVR rate was 51.7%. Biopsy fibrosis scores were available for 95% of the patients in this group, and the majority of those exhibited fibrosis stage 4. The inclusion criteria for study subjects were to be infected with HCV genotype 1, and to be over 20 years of age. The study was approved by the Stockholm Regional Ethics Committee (approval number 2012/63-31/1), and informed consent was provided by all participants according to the Declaration of Helsinki. For the majority of patients in the triple therapy cohort written consent was obtained. For a minority of patients in the triple therapy cohort, and for all dual therapy patients, oral consent was obtained and documented in each patient’s journal in accordance with the rules of the Stockholm County Clinical Biobank at the Karolinska University Hospital. The oral consent procedure is generally used for patients who are followed regularly at the infectious disease clinic and donate blood samples for biobanking for immunological studies. This consent procedure was approved by the Stockholm Regional Ethics Committee.

**Table 1:** Patient data for the dual and triple therapy cohorts.

	Dual therapy cohort	Triple therapy cohort
Subjects	72	89
SVR	45 (62.5%)	46 (51.7%)
Sex		
Male	51 (71%)	59 (66%)
Female	21 (29%)	30 (34%)
HCV gt1 subtype		
a	57 (79.2%)	70 (78.7%)
b	14 (19.4%)	17 (19.1%)
c	1 (1.4%)	nd
Viral load		
Median	1,900,000	24,562
Range	3,400–69,000,000	4,990–9,930,000
Fibroscan scores		
n	19	71
Liver median (range)	6.8 (3.7–27)	13.9 (4.2–75)
Biopsy scores		
Fibrosis, n	49	85
0	1	1
1	12	9
2	16	13
3	15	17
4	5	45

Abbreviations: SVR, sustained virological response; nd, no data.

### ELISA assays

Plasma levels of IL-18 and sCD14 were measured by ELISAs from MBL (Japan) and R&D Systems (USA), respectively, and performed according to manufacturers’ instructions with fluorescence intensity measured using a Bio-Rad iMark microplate reader.

### Statistical analysis

All statistical analysis was performed using Graph Pad Prism version 5.0a for Mac OSX (GraphPad Software, La Jolla, CA). Gaussian distribution of populations was evaluated using D’Agostino and Pearson omnibus normality test. Direct comparisons between two groups were done using either an unpaired T-test with Welch’s correction if required, or the Mann-Whitney test. Repeated measures ANOVA or Friedman test were used for longitudinal comparisons of patients over the course of treatment. The chi-squared test for trend was used when comparing the frequency distribution of patient outcomes in quartiles of analytes measured. P values<0.05 were considered statistically significant.

## Results

### Elevation of plasma sCD14 and IL-18 levels during interferon-based treatment

**Figure 1:**
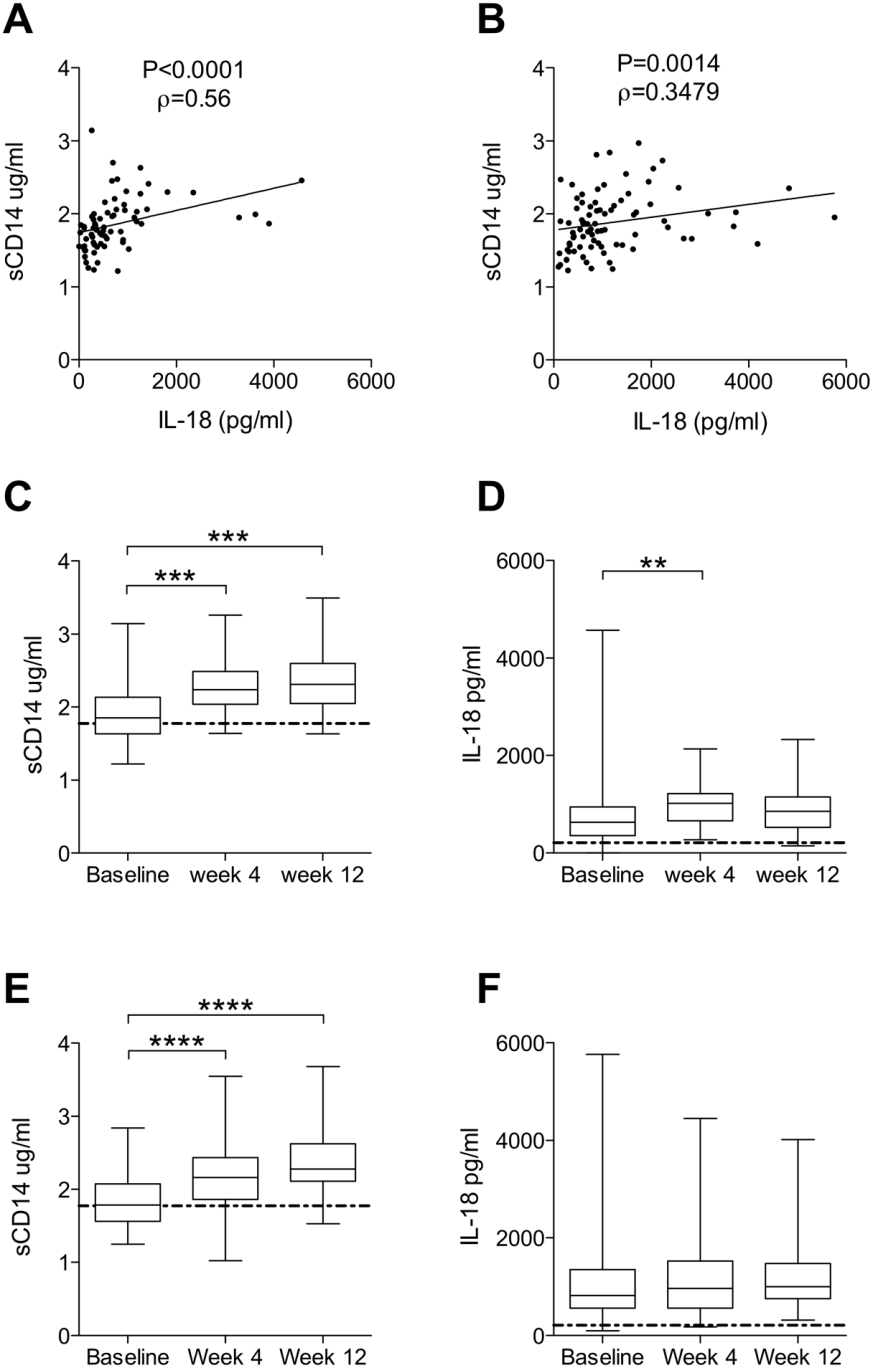
Dynamics of sCD14 and IL-18 over the course of dual and triple therapy. (A) Correlation of baseline levels of sCD14 and IL-18 in dual therapy patients (n = 72, Spearman rho = 0.5587, P<0.0001). (B) Correlation of baseline levels of sCD14 and IL-18 in triple therapy patients (n = 82, Spearman rho = 0.3479, P = 0.0014). (C) sCD14 over the course of dual therapy (n = 50, Friedman test P<0.0001). (D) IL-18 levels during dual therapy (n = 50, Friedman test P = 0.0019). (E) sCD14 over the course of triple therapy (n = 53, repeated measures ANOVA P<0.0001). (F) IL-18 levels during treatment with triple therapy (n = 53). *P≤0.05, **P≤0.01, ***P≤0.001, ****P≤0.0001 (for Dunn’s comparison or Bonferroni’s comparison were appropriate); dotted line represents untreated healthy control.

Given the role of sCD14 and IL-18 as markers and mediators of the innate immune response, we were interested to evaluate their plasma levels as candidate biomarkers of HCV treatment effects and outcome. sCD14 and IL-18 were measured in plasma samples drawn at treatment baseline, at 4 weeks of treatment, and at 12 weeks of treatment, and the dynamics of these proteins were analyzed. Baseline levels of sCD14 and IL-18 were positively correlated in both patient cohorts (Figure 1A–B, dual therapy rho = 0.5587, P<0.0001; triple therapy rho = 0.3479, P = 0.0014). In patients undergoing dual therapy, levels of both sCD14 and IL-18 were significantly increased at week 4 (P≤0.001 and P≤0.01, respectively) (Figure 1C–D), with the increase of sCD14 persisting to week 12 (P≤0.001). Next, sCD14 and IL-18 dynamics were analyzed in patients undergoing triple therapy with peg-IFNα, ribavirin and Telaprevir. sCD14 was increased 4 weeks after initiation of therapy (P≤0.0001), and this elevated level persisted to week 12 (P≤0.0001) (Figure 1E). Unlike the pattern observed in patients undergoing dual therapy however, IL-18 displayed no such increase in triple-therapy patients (Figure 1F).

### Altered dynamics and higher baseline levels of sCD14 and IL-18 in patients whom do not respond to therapy

**Figure 2:**
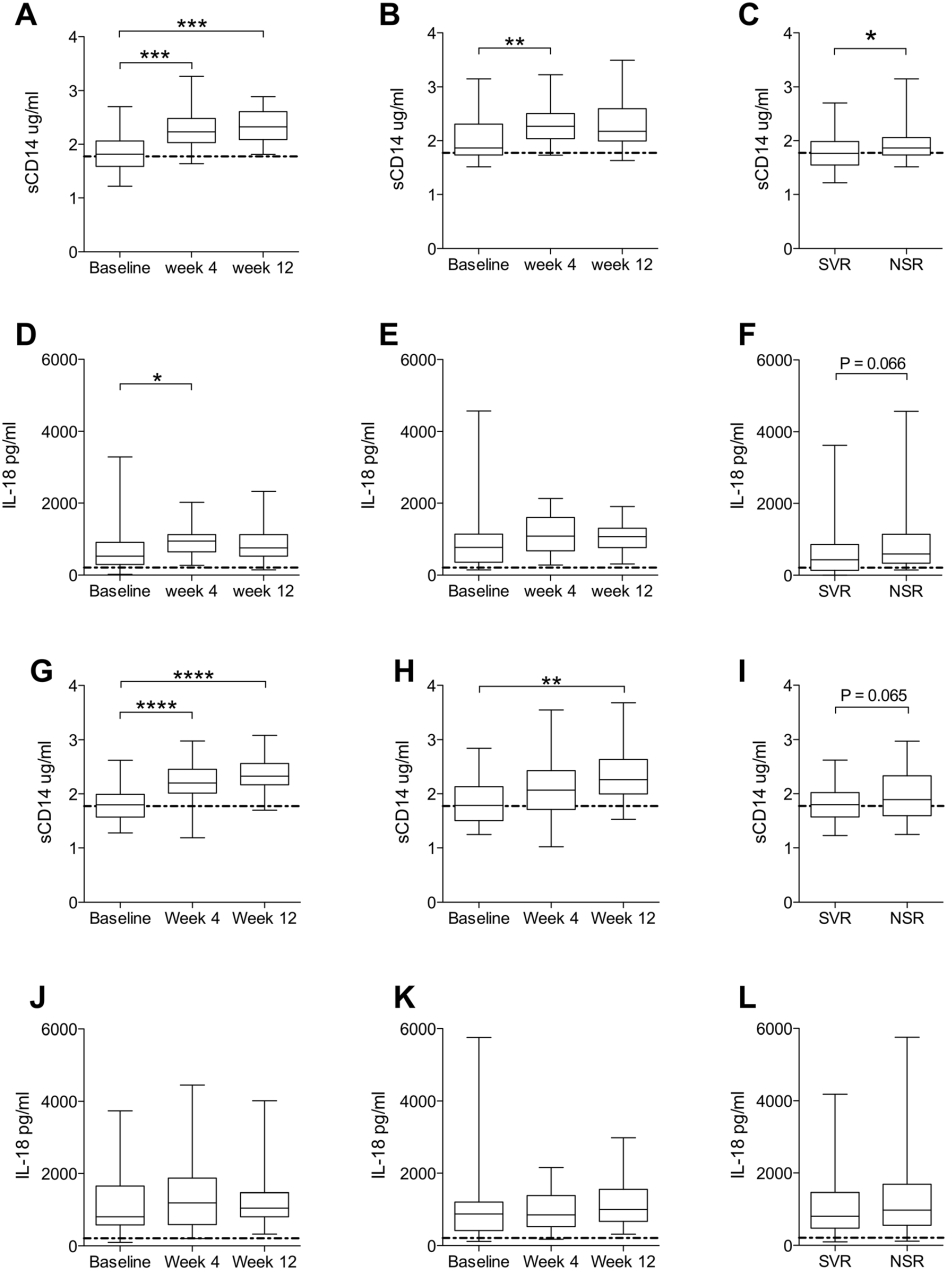
Dynamics of sCD14 and IL-18 are differentially associated with outcome during dual and triple therapy. (A) sCD14 levels in patients reaching a sustained virological response (SVR) (n = 31, Friedman test P<0.0001), and (B) non-SVR (NSR) patients (n = 19, Friedman test P = 0.0044). (C) Comparison of baseline sCD14 between SVR and NSR patients (SVR n = 45, NSR n = 27, Mann Whitney P = 0.0348). (D) IL-18 levels in SVR patients (n = 31, Friedman test P = 0.0183), and (E) NSR patients (n = 19, Friedman test P = 0.104). (F) Comparison of baseline IL-18 between SVR and NSR patients (SVR n = 45, NSR n = 27, Mann Whitney P = 0.0661). Panels (A–F) depict data from dual therapy cohort. (G) sCD14 levels in SVR patients (n = 26, repeated measures ANOVA P<0.0001), and (H) NSR patients (n = 27, repeated measures ANOVA P = 0.0017). (I) Comparison of baseline sCD14 between SVR and NSR patients (SVR n = 42, NSR n = 40, unpaired T test with Welch’s correction P = 0.0647). (J) IL-18 levels in SVR patients (n = 26), and (K) NSR patients (n = 27). (L) Comparison of baseline IL-18 between SVR and NSR patients (SVR n = 42, NSR n = 40). Panels (G–L) depict data from triple therapy cohort. *P≤0.05, **P≤0.01, ***P≤0.001, ****P≤0.0001 (for Dunn’s comparison, Bonferroni’s comparison, or Mann Whitney were appropriate); dotted line represents untreated healthy control.

Patients were subdivided into groups that subsequently went on to obtain a sustained virological response (SVR) or no sustained response (NSR) to treatment (Figure 2). Increases of sCD14 in the dual therapy cohort were less clear in NSR patients compared to SVR patients, with increases in sCD14 at week 12 no longer significant and a weaker significance at week 4 (Figure 2A–B). Similarly, IL-18 levels were elevated at week 4 in SVR patients (P≤0.05), but this was not the case in NSR patients (Figure 2D–E). Baseline levels of sCD14 were significantly lower in patients whom achieved a SVR (P = 0.0348) (Figure 2C), while IL-18 displayed a similar trend (P = 0.066) (Figure 2F). In the triple therapy cohort the increase of sCD14 was preserved in the SVR group, while it was lost in the NSR group at week 4 and only modestly increased at week 12 (P≤0.01) (Figure 2G–H). IL-18 displayed no apparent differences between SVR and NSR patients after therapy initiation for the triple therapy cohort (Figure 2J–K). Baseline levels of sCD14 displayed a trend towards lower levels in patients whom responded to treatment (P = 0.065) (Figure 2I), while there was no such trend observed for IL-18 (Figure 2L).

### Combination of low sCD14 and low IL-18 plasma baseline levels predict treatment outcome in dual therapy

**Figure 3:**
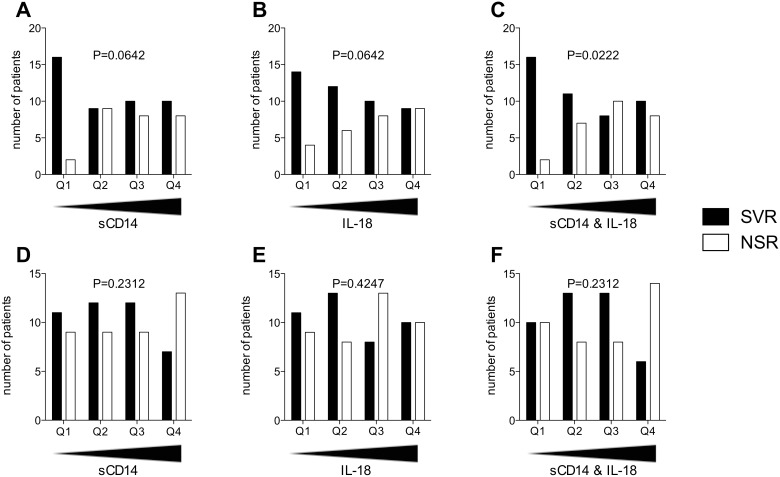
Associations between treatment outcome and levels of sCD14 and IL-18 in plasma: effect of Telaprevir. Patients were ranked according to their baseline levels of sCD14, IL-18 or a combination of the two, ((x–x)/IQRx)+((y–y)/IQRy), from lowest to highest, with number of patients with a sustained virological response (SVR) or no sustained response (NSR) in each quartile assessed. (A) Number of SVR and NSR patients for increasing levels of baseline sCD14 in patients receiving dual therapy (n = 72). (B) Number of SVR and NSR patients for increasing levels of baseline IL-18 in patients receiving dual therapy (n = 72). (C) Number of SVR and NSR patients for increasing levels of the combination of the two analytes in patients receiving dual therapy (n = 72). (D) Number of SVR and NSR patients for increasing levels of baseline sCD14 in patients receiving triple therapy (n = 82). (E) Number of SVR and NSR patients for increasing levels of baseline IL-18 in patients receiving triple therapy (n = 82). (F) Number of SVR and NSR patients for increasing levels of the combination of the two analytes in patients receiving triple therapy (n = 82). P values shown represent Chi-squared test for trend.

To better understand the associations between innate immune biomarkers and treatment outcome in more detail, patient quartiles based on plasma levels of sCD14 and IL-18 at baseline were analyzed for their treatment response rates (Figure 3). For sCD14 the SVR rate of the patients with the lowest values in quartile 1 (Q1) was 89%, whereas the response rate of the other quartiles ranged from 50% to 56% (P = 0.0642) (Figure 3A). IL-18 displayed a more gradual relationship with the SVR rate dropping sequentially from 78% in Q1 to 50% in Q4 (P = 0.0642) (Figure 3B). As both sCD14 and IL-18 showed trends toward an association with treatment response, the two analytes were combined together using normalized values, relative to their interquartile range, ((x–x)/IQRx)+((y–y)/IQRy), to establish if a higher SVR rate could be predicted. Using this combination score we observed that the lowest quartile of the combined sCD14 and IL-18 normalized levels predicted an SVR rate of 89% (P = 0.0222) (Figure 3C).

### Telaprevir alters associations between plasma levels of sCD14 and IL-18, and treatment outcome

A different pattern emerged when examining the triple therapy outcome data in relation to sCD14 and IL-18 baseline level quartiles. For sCD14 the SVR rate for Q1 to Q3 remained steady between 55% and 57%, falling to 35% in Q4 (P = 0.2312) (Figure 3D). IL-18 showed no discernable pattern in relation to the triple therapy response rate (Figure 3E). For the triple therapy patients, the combination of sCD14 and IL-18 was in line with the sCD14 data beginning at a 50% SVR rate for Q1, a moderate rise to 61% for Q2 and Q3, and finally dropping to 30% in Q4 (P = 0.2312) (Figure 3F). Thus, the addition of Telaprevir to the treatment regimen abolishes the sCD14 and IL-18 combination score prediction of outcome.

### Differential dynamic ranges of increase in sCD14 in response to treatment

**Figure 4:**
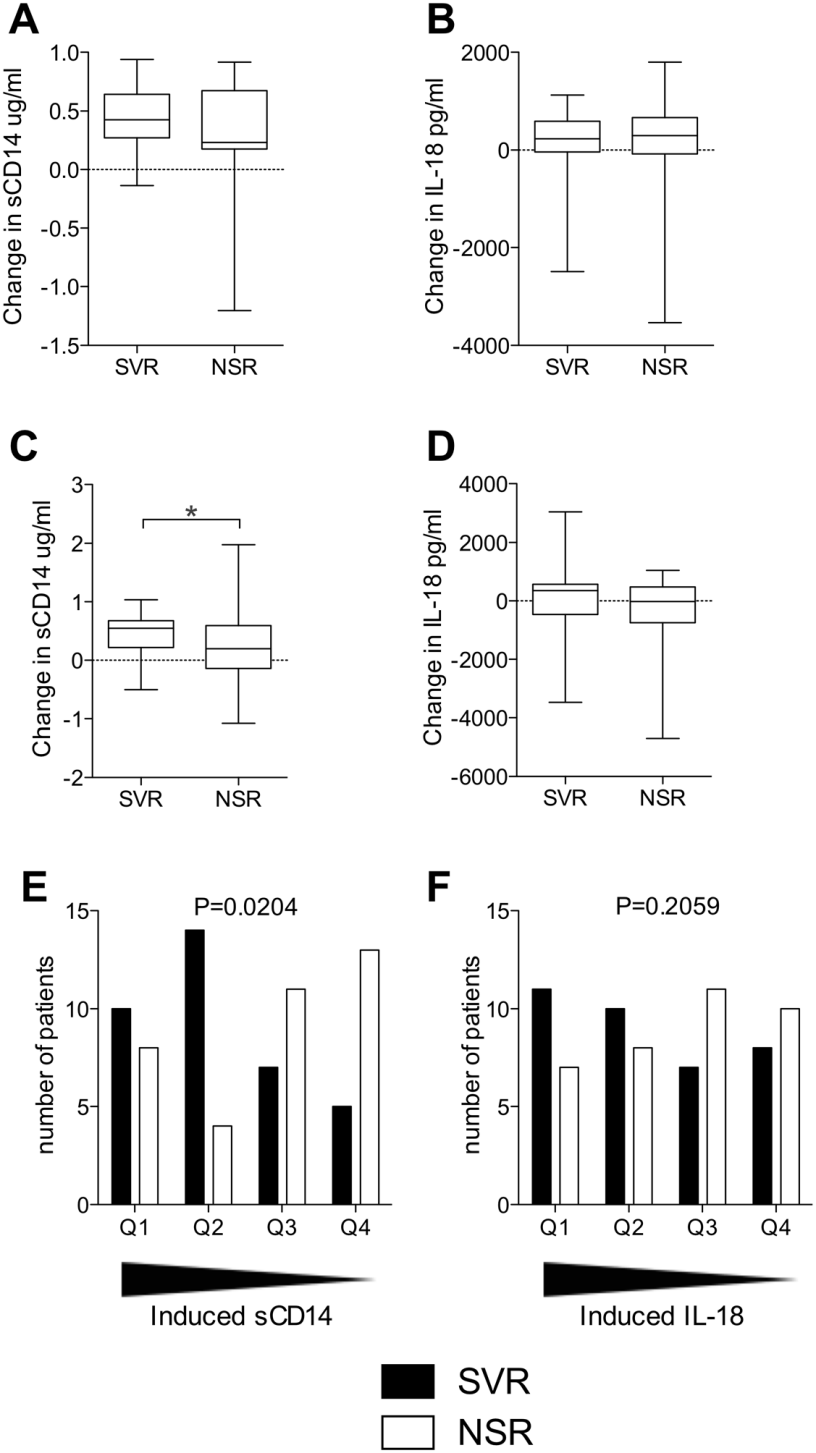
Induced elevation of sCD14 is associated with triple therapy outcome. (A) Differences in the induction of sCD14 between sustained virological response (SVR) and no sustained response (NSR) patients whom undergo dual therapy (SVR n = 33, NSR n = 19). (B) Differences in the induction of IL-18 between SVR and NSR patients whom undergo dual therapy (SVR n = 33, NSR n = 19). (C) Differences in the induction of sCD14 between SVR and NSR patients whom undergo triple therapy (SVR n = 36, NSR n = 36, unpaired T test with Welch’s correction P = 0.0435). (D) Differences in the induction of IL-18 between SVR and NSR patients whom undergo triple therapy. *P≤0.05. Patients in the triple therapy cohort were ordered with those with the highest levels of sCD14 elevation in quartile 1 (Q1) to the lowest in Q4 and number of patients with a SVR or NSR in each quartile was assessed. (E) Number of SVR and NSR patients in each sCD14 quartile (n = 72). (F) Number of SVR and NSR patients in each IL-18 quartile (n = 72). P values shown represent Chi-squared test for trend.

Following on from the observed dynamics of the two analytes we assessed their change in response to the two regimens after 4 weeks of treatment in relation to treatment outcome. SVR and NSR groups did not differ with regard to treatment-induced change of sCD14 or IL-18 in the dual therapy cohort (Figure 4A–B). In the triple therapy cohort we observed a greater increase of sCD14 in response to therapy in patients that experienced a SVR (P = 0.0435), while no such effect was noted for IL-18 (Figure 4C–D). As an increase in sCD14 levels was observed in response to triple therapy, we divided these patients into quartiles of magnitude of sCD14 increase with the highest increases in Q1. The SVR rates were 56% and 78% for Q1 and Q2 respectively, whereas they declined to 39% and 28% in Q3 and Q4 respectively (P = 0.0204) (Figure 4E). Although a similar pattern was observed for IL-18 with the SVR rate beginning at 61% in Q1 and falling to 44% in Q4, this did not reach statistical significance (Figure 4F). These data indicate that the dynamic increase in sCD14 is a candidate biomarker of outcome in patients undergoing triple therapy with peg-IFNα, ribavirin and Telaprevir.

## Discussion

Soluble CD14 and IL-18 are markers and mediators of the innate immune response, and their plasma levels are candidate biomarkers of HCV treatment effects and outcome. In the present study, we show that sCD14 dynamics has prognostic value in predicting outcome of peg-IFNα and ribavirin therapy in HCV genotype 1 infection. Combining sCD14 with IL-18 strengthens this association, whereas the association is markedly weaker in patients whom receive Telaprevir in addition to peg-IFNα and ribavirin. In the triple-therapy setting it is instead the dynamic increase in sCD14 induced by treatment that is associated with a positive treatment outcome. Inclusion of a DAA thus appears to change the dynamic relationship between innate immune biomarkers and interferon-based treatment effects and outcome.

Patients that clear HCV in response to standard peg-IFNα and ribavirin therapy have low baseline levels of sCD14 in plasma. These results confirm a pattern previously observed in HCV/HIV-1 co-infected patients undergoing the same treatment for their HCV infection [21], [22]. Furthermore, our detailed analysis show that the lowest levels are associated with a very high SVR rate, with 89% of patients in the lowest quartile clearing the virus, and all patients with sCD14 levels lower than 1.5 µg/ml at baseline (n = 9) achieving a SVR. It thus appears that it is not high sCD14 values that are associated with poor response to treatment, but rather the very lowest levels that are associated a high response rate. For IL-18 the pattern is somewhat different in that the plasma concentration shows a more gradual association with the treatment response rate, with lower levels indicating a more favorable outcome. A normalized combination score of these analytes strengthened this association between plasma levels and the likelihood of a SVR in response to dual therapy. Intriguingly, this combinatorial effect was not evident in the triple therapy cohort.

Peg-IFNα-based dual therapy caused distinct increases in levels of sCD14 and IL-18, and these effects were stronger in patients whom went on to clear the infection. Both these proteins can therefore be viewed as interferon-induced biomarkers. However, the increase of these analytes between baseline and week 4 of therapy was not associated with the likelihood of clearing the virus. Instead it appears that the pre-treatment baseline values of sCD14 and IL-18 are more significant in this regard. However, with the addition of Telaprevir to the treatment regimen, the increase in sCD14 on treatment was associated with treatment outcome rather than the initial baseline level. This difference may be due to the effect of Telaprevir restoring sensitivity to the peg-IFNα therapy as previously suggested [10].

IL-18 has been extensively studied and is known to be involved in responses to viral infections [24], hepatocyte apoptosis [25], and linked to chronic liver damage [26], [27]. The specific origin of sCD14 in HCV infection, what it indicates about the progression of the disease, and hence the basis for its role as a predictor of SVR rate is still to be elucidated. There are many aspects to consider when deliberating the source of sCD14 in the HCV setting, including microbial translocation, and liver function [15], [18]. Although we did not see a correlation between sCD14 and fibrosis stage as has been described elsewhere [13], it is possible the sCD14 is a marker of liver inflammation and damage. Elevated sCD14 may also indicate an increased state of innate immune activation associated with monocyte and Kupffer cell activation [12], [15]. This possibility is also supported by the correlation between sCD14 and IL-18 levels observed in the present study. Indeed it may be that low sCD14 and IL-18 levels are indicative of a low level of activation of IFN-stimulated genes, a pattern that was previously linked to the outcome of IFNα-based therapies in HCV infection [2], [3], [30]. This notion is supported by our observation that sCD14 levels in plasma increase quite significantly during IFNα therapy.

Given these different possibilities on the origins of sCD14, it would be useful to examine the mechanisms governing its production in various liver diseases, to help further understand its role as a biomarker. As new DAAs against HCV are beginning to be prescribed without the IFNα backbone [31]–[33], it would also be interesting and useful to study the role sCD14 plays in the new standard of care. In conclusion, IFNα and ribavirin therapy of HCV infection increases innate immune activation as measured by sCD14 and IL-18. The lowest baseline levels of these analytes in combination, rather than their increase upon therapy, identify a group of patients with a very high SVR rate. Telaprevir appears to abrogate this relationship, allowing patients with higher baseline sCD14 to respond to therapy. Within this setting it is instead the dynamic increase of sCD14 which associates with a favorable response to treatment.
